# Near complete response recurrent glioblastoma after treatment with [^131^I]-Iodofalan

**DOI:** 10.1007/s00259-025-07742-w

**Published:** 2026-01-14

**Authors:** A. El Ghalbouni, T. J. Snijders, N. Tolboom, A. J. A. T. Braat

**Affiliations:** 1 Radiology and Nuclear Medicine, UMC Utrecht, Utrecht, the Netherlands; 2 Neurology and Neurosurgery, Brain Center, UMC Utrecht, Utrecht, the Netherlands; 3 Nuclear Medicine, Netherlands Cancer Institute, Amsterdam, the Netherlands; 4 UMC Utrecht, Heidelberglaan 100, Utrecht, 3584 CX Netherlands

Glioblastoma is an incurable disease with 5-year survival rates below 10%. Despite advances in molecular diagnostics and subtyping of these high-grade tumors, an effective treatment to replace temozolomide-based chemoradiation as the standard of care has yet to emerge. One promising therapy under investigation is Iodofalan (^131^I) ([^131^I]IPA), a form of radionuclide therapy [1].

A 56-year-old man presented with progressive, treatment-refractory glioblastoma (WHO grade 4, IDH-wildtype, with methylated MGMT promoter) that diffusely infiltrated the left parietotemporal lobes, causing dysarthria and hemifacial spasms. After biopsy, treatment consisted of temozolomide-based chemoradiation and adjuvant cycles of adjuvant temozolomide. Twelve months after initial presentation, following suspected tumor progression on MRI, subsequent [^18^F]FET-PET/CT confirmed progression. Neurosurgeons deemed the recurrence inoperable due to its location. As the standard-of-care options were limited, intravenous treatment with [^131^I]IPA in compassionate use was considered. Treatment was initiated with 2 cycles of 5 GBq per 4 weeks interval. Consequently, due to delivery problems, 3 cycles of lomustine were given as bridging therapy, but lomustine had to be discontinued due to pancytopenia. Two additional cycles of 5 GBq [^131^I]IPA were given. The patient tolerated all [^131^I]IPA cycles well and showed improvement of all neurologic deficits over time, with decrease in dysarthria and improved overall wellbeing. Tumor response on serial MRI every 3 months and [^18^F]FET-PET/CT every 2 months demonstrated ongoing regression of the residual enhancing, [^18^F]FET-positive tumor (partial response according to PET RANO criteria) [2]. To date, 31 months after initial biopsy (23 months after starting [^131^I]IPA) his treatment response is ongoing. Pancytopenia has completely recovered during follow-up and no side effect or long-term complications have been observed to date (Fig. 1).

**Fig. 1:**
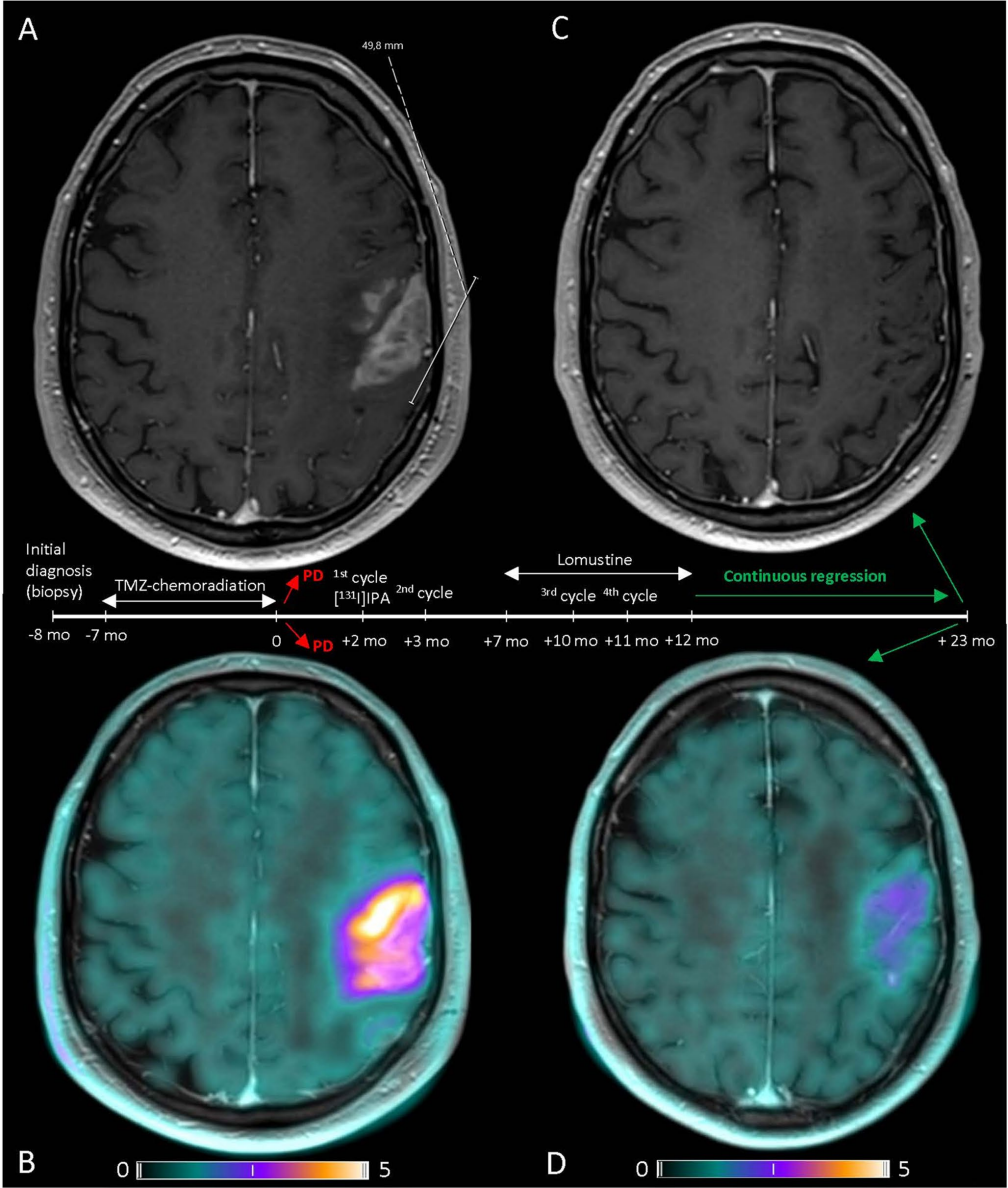
Timeline and imaging response of recurrent glioblastoma after combined [^131^I]IPA and lomustine. **A** Pre-[^131^I]IPA gadolinium-enhanced T1-weighted MRI (gdMR) demonstrating recurrence of a glioblastoma in the left parietotemporal lobe with a maximum diameter of 49 mm. **B** Pre-[^131^I]IPA [^18^F]FET-PET/CT revealing pathological uptake with a maximum tumor-to-background ratio (TBRmax) of 5.6, confirming tumor recurrence and validating the patient’s eligibility for treatment with [^131^I]IPA. **C** Follow-up gdMR performed 23 months (mo) after having completed 4 cycles of [^131^I]IPA and 3 cycles of lomustine, showing near-complete resolution of contrast enhancement. **D** Follow-up [^18^F]FET-PET/CT demonstrating significantly decreased pathological uptake with a residual TBRmax of 2.8, consistent with a partial response 23 months after initiating [^131^I]IPA treatment

Prospective studies are currently ongoing with [^131^I]IPA as a concomitant treatment with first-line chemoradiation for glioblastoma. This promising case warrants validation in prospective studies of [^131^I]IPA, possibly with chemotherapy, for recurrent [^18^F]FET-positive high-grade glioma.

## Data Availability

All relevant data supporting the findings of this case report are included within the manuscript.
